# Case Report: Recurrent intrahepatic cholestasis: two rare cases with their novel variants of ATB8B1 and atypical clinical findings

**DOI:** 10.3389/fmed.2026.1886444

**Published:** 2026-07-10

**Authors:** Jiaxun Li, Qi Wei, Sicong Liu, Luyu Lv, Huarong Ding, Liping Guo, Diefei Hu, Qiuyue Ning

**Affiliations:** 1 Department of Microbiology, Guangxi Medical University, Nanning, Guangxi, China; 2 The First Affiliated Hospital of Guangxi Medical University, Nanning, Guangxi, China; 3 Department of Infectious Diseases, The First Affiliated Hospital of Guangxi Medical University, Nanning, Guangxi, China; 4 Guangxi Key Laboratory of Thalassemia Research, Guangxi Medical University, Nanning, Guangxi, China; 5 Department of Burns and Plastic Surgery, Guangxi Medical University, Nanning, Guangxi, China; 6 Key Laboratory of Basic Research on Regional Diseases (Guangxi Medical University), Education Department of Guangxi Zhuang Autonomous Region, Nanning, Guangxi, China

**Keywords:** ATP8B1 gene, autosomal recessive, case report, compound heterozygous variants, recurrent intrahepatic cholestasis

## Abstract

Recurrent intrahepatic cholestasis type 1 (RIC1), historically also known as BRIC 1 (benign recurrent intrahepatic cholestasis typr 1),is an autosomal recessive disorder presenting with intermittent episodes of cholestatic jaundice and caused by pathogenic variants of adenosine triphosphatase phospholipid transporting 8B1 (ATP8B1). Here, we describe two unusual RIC1-affected siblings, whose clinical courses and findings deviate from the classical RIC1 patients, emphasizing the potential adverse effects associated with recurrent cholestasis. Additionally, we have for the first time presented specific genetic, clinical and pathological evidence demonstrating that two novel compound heterozygous variants of ATP8B1 (c.749 T > C and c.3261 + 5G > A) had a significant effect on the expression of the ATP8B1 encoded protein, thereby contributing to cholestasis attacks.

## Introduction

Familial intrahepatic cholestasis can be divided into three groups, namely Recurrent intrahepatic cholestasis (RIC), progressive familial intrahepatic cholestasis (PFIC) and intrahepatic cholestasis of pregnancy. Of which RIC includes RIC types 1 and 2. The two types are due to pathogenic variants in adenosine triphosphatase phospholipid transporting 8B1 [ATP8B1, a gene encodes familial intrahepatic cholestasis 1 (FIC1) protein] and ABCB11 [Adenosine triphosphate binding cassette transporter superfamily, subfamily B, member 11, which encodes bile salt export pump (BSEP)], respectively (1, 2). In patients with FIC1 or BSEP deficiency, the asymmetry of aminophospholipids in the hepatocanalicular membrane is disrupted, and the transport activity of the bile acid (BA) transport pump is reduced, thus lead to hepatocyte BA overload and are involved in ranging from mild to severe or benign phenotypes (3–5).

RIC is usually benign with self-limited recurrent attacks of jaundice, pruritus, and heals without significant liver damage. The initial attack of RIC tends to occur within the first two decades of a patient’s life (6). Attacks can occur unprompted but can often be precipitated by infections or pregnancy (7). Cholestasis attacks are of similar character, however, the duration and severity of these attacks, and asymptomatic intervals between each attack vary considerably among patients (8). During the attacks in these patients, serum bilirubin values, alkaline phosphatase (ALP) and total bile acid (TBA) are markedly elevated, whereas gamma-glutamyl transpeptidase (GGT) levels are mostly normal or slightly elevated. Histopathological examination of liver biopsy specimens frequently reveals central lobular cholestasis (9).

This article reports the clinical management of two siblings diagnosed with RIC1 and delineates the ATP8B1 variant carrier status among other key family members. These two unique cases enhance our understanding of the atypical findings observed in RIC1 patients. Moreover, lessons from the treatment outcome of Case 1 and valuable references for the potential effects of two novel ATP8B1 variants identified in Case 2 were gained from our data.

## Case report

### Case 1

A 35-year-old man was admitted to a local hospital with jaundice, pruritus and clay-colored stools. These symptoms started when he was 3 years old and recurred intermittently, occurring a total of six times. Prior to the preceding three attacks, he had received antibiotic treatment (the specific antibiotics used were unknown). He had no other risk factors for liver injury, such as blood transfusions, alcohol intake, and inborn or acquired metabolic etiologies. One of his sisters exhibits similar symptoms as him (Case 2), while the other family members remain asymptomatic.

The details regarding his clinical visits during the first five attacks remain undocumented. Laboratory data from his sixth visit to the local hospital are as follows: serum total bilirubin, 824.1 μmol/L (ref < 17.0 μmol/L); serum direct bilirubin, 531.7 μmol/L (ref 1.7–6.9 μmol/L); serum indirect bilirubin, 292.4 μmol/L (ref < 12 μmol/L); total protein, 49.1 g/L (ref 65–85 g/L); albumin, 35.4 g/L; total bile acid, 266.1 μmol/L (ref < 12 μmol/L); alkaline phosphatase (ALP), 270 U/L (ref < 130 U/L); gamma-glutamyl transpeptidase (GGT) activity, 125 U/L (ref < 50 U/L); serum aspartate aminotransferase (AST), 46 U/L (ref < 40 U/L); and alanine aminotransferase (ALT), 99 U/L (ref < 40 U/L). Negative results were found for the following parameters: IgM antibodies to hepatitis A virus, hepatitis B surface antigen, antibodies to hepatitis C virus and hepatitis E virus, cytomegalovirus antibody, Epstein–Barr virus antibody, antinuclear antibodies, autoimmune hepatitis antibody profile, serum copper and ceruloplasmin levels, and immunoglobulins (IgG, IgA, and IgM).

**Figure 1:**
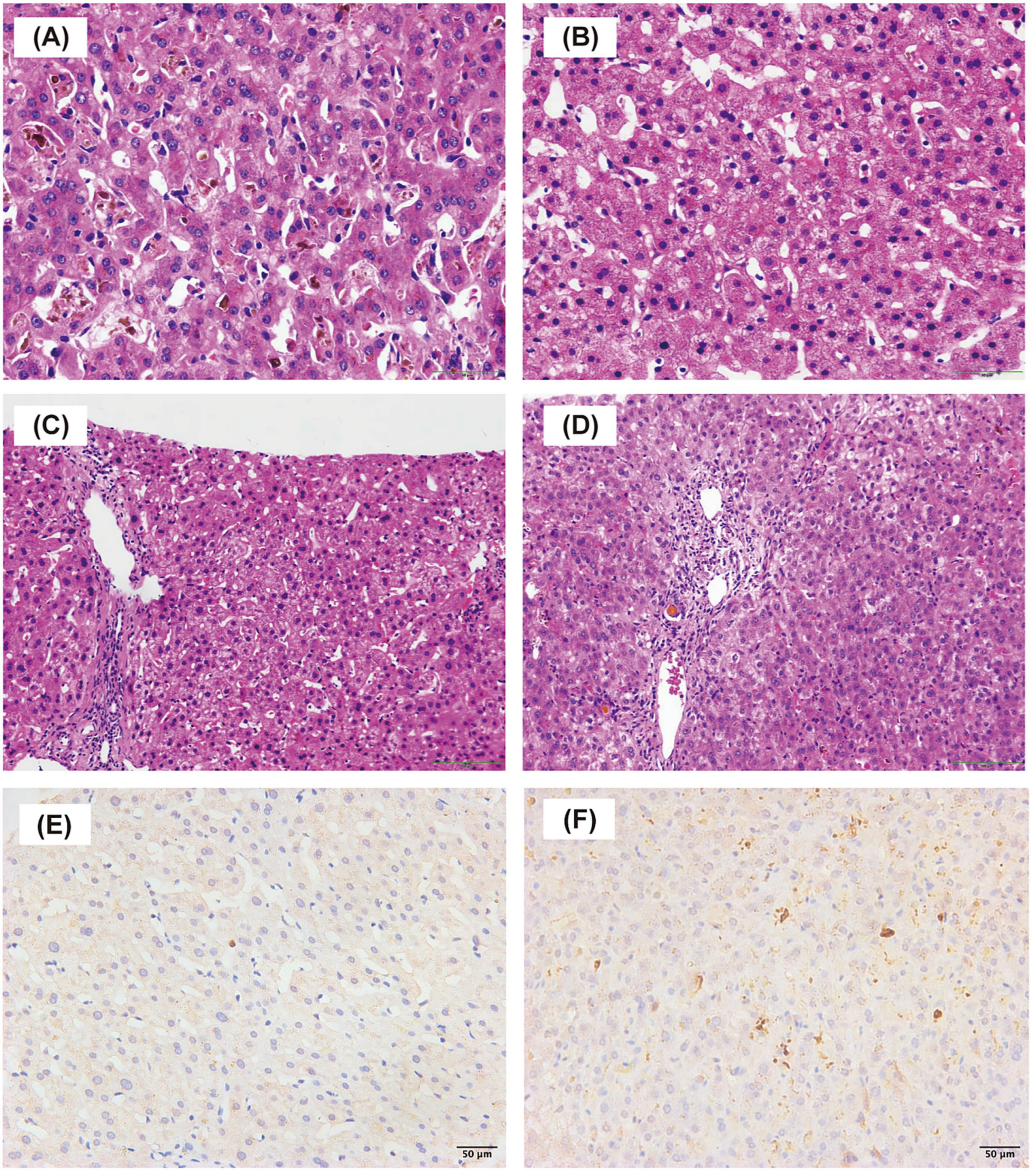
Histopathologic findings in Case 1 **(A,B)** and Case 2 **(C,D)**, as well as Immunohistochemistry for ATP8B1 gene encoded protein of Case 2 **(E,F)**. **(A)** Liver biopsy showed preserved biliary ducts, cholestasis in zone 3 (centrilobular area) with evidence of bile plugs, intra-hepatocyte bile pigment and mild fibrosis in the portal areas [hematoxylin and eosin (HE), 200 ×]. **(B)** Spotty necrosis accompanied by few inflammatory cells were observed in the peripheral regions of the hepatic lobules (HE, 200 ×). **(C)** Liver tissue showed preserved hepatic lobular architecture with well-organized hepatocytes, accompanied by cholestasis and spotty necrosis in centrilobular area (HE, 100 ×). **(D)** Portal areas showing preserved biliary duct and mild fibrous tissue proliferation accompanied by few inflammatory cells (HE, 100 ×). **(E)** Canalicular immunoreactivity for ATP8B1 gene encoded protein showing mild or absent staining in the liver tissue of Case 2 (200 ×). **(F)** Normal immunohistochemical ATP8B1 gene encoded protein staining in a healthy control (200 ×).

The abdominal ultrasound revealed no significant abnormalities. Magnetic resonance imaging (MRI) of the liver and gallbladder, including non-contrast imaging, contrast-enhanced imaging, and MR cholangiopancreatography, demonstrated mild hepatosplenomegaly along with normal intra and extra-hepatic biliary ducts and pancreatic ductal system. No evidence of portal hypertension was detected. The liver biopsy revealed cholestasis in the centrilobular hepatocytes, with evidence of bile plugs, intra-hepatocyte bile pigment and mild fibrosis in the portal areas. Spotty necrosis accompanied by few inflammatory cells were observed in the peripheral regions of the hepatic lobules (Figures 1A,B).

Following the acquisition of informed consent, targeted next-generation sequencing, involving 61 genes responsible for genetic disorders of hepatic cholestasis (10), was performed on him to further clarify the diagnosis. The genetic analyses revealed seven variants of ATP8B1 (Supplementary Table 1 and Supplementary Figures 1A–G). Thus, he was highly suspected to have RIC1 owing to these clinical, genetic, and pathological findings.

He was treated with hepatoprotective agents and medications to relieve cholestasis, including anethol trithione capsules and ursodeoxycholic acid; Nevertheless, these interventions were ineffective in reducing the bilirubin levels. In consideration of his hepatosplenomegaly, subsequent treatment was initiated with a combination of glucocorticoids and azathioprine (the specific dosages of these medications remain undocumented). Following drug administration, a rapid decline in white blood cell count was observed. The patient succumbed to sepsis several days later.

### Case 2

Case 2 is the sister of Case 1 and is 30 years old. She was admitted to our hospital with jaundice, pruritus, dark urine, and clay-colored stools. These symptoms started when she was 10 years old and recurred intermittently thereafter. Upon admission, this was the fourth attack, with symptoms predominantly occurring following exhaustion. During each attack, the patient experienced digestive symptoms including loss of appetite, nausea, and vomiting. On the first three attacks, the local hospital provided hepatoprotective agents, jaundice-lowering therapy and hormonal therapy (The specific medications included ursodeoxycholic acid capsules, compound glycyrrhizin tablets, and prednisone tablets; however, the dosage information was unavailable), resulting in symptomatic improvement. Due to recurrence of the condition, the patient presented to our hospital for further evaluation and management. No fever or infections were reported. The patient had no known risk factors for liver injury, and her bilirubin, transaminases, GGT, bile acids and ALP levels remained within normal limits during the asymptomatic intervals.

On physical examination, the patient showed an evident jaundice, without clinical signs of ascites, edema, and hepatic encephalopathy. Laboratory data demonstrated that the levels of total bilirubin, direct bilirubin, total bile acids, ALT, AST, and ALP were markedly elevated, whereas GGT activity remained within the normal reference range. The negative results were consistent with that of her brother, as previously noted. Abdominal ultrasound and contrast-enhanced magnetic resonance imaging revealed no significant abnormalities with intra and extra-hepatic biliary ducts. The liver biopsy revealed a preserved hepatic lobular architecture with well-organized hepatocytes, accompanied by cholestasis and spotty necrosis in centrilobular area. Mild fibrous tissue proliferation and few inflammatory cells were observed in the portal areas (Figures 1C,D).

Considering her family history of IRC1, after obtaining informed consent, a genetic examination was performed to confirm the clinical diagnosis. Targeted next-generation sequencing detected two heterozygous variants of ATP8B1: c.749 T > C and c.3261 + 5G > A (Supplementary Table 1 and Supplementary Figures 1H,I), consistent with the autosomal recessive inheritance pattern characteristic of RIC. Immunohistochemistry studies were conducted to further assessed the impact of ATP8B1 variants on protein expression levels, indicating that the ATP8B1 gene encoded protein (anti-ATP8B1 Antibody, rabbit polyclonal antibody HPA018673, 1:50 working dilution, Atlas Antibodies, Stockholm, Sweden. This antibody has a specific affinity for the protein codified by ATP8B1 gene) expression in the liver of Case 2 was significantly reduced as compared to that of a healthy control (Figures 1E,F).

She was treated with ursodeoxycholic acid (UCDA) capsules 250 mg three times daily. On the 11th day of admission, liver function tests were repeated, showing a reduction in ALT and AST levels to 38 U/L and 30 U/L, respectively; however, total bilirubin increased to 101.10 μmol/L. Consequently, prednisone tablets 20 mg were initiated orally once daily. On the 17th day of admission, follow-up liver function testing revealed a decline in total bilirubin to 60.40 μmol/L, with marked improvement in jaundice and pruritus. The patient was discharged the following day.

**Figure 2:**
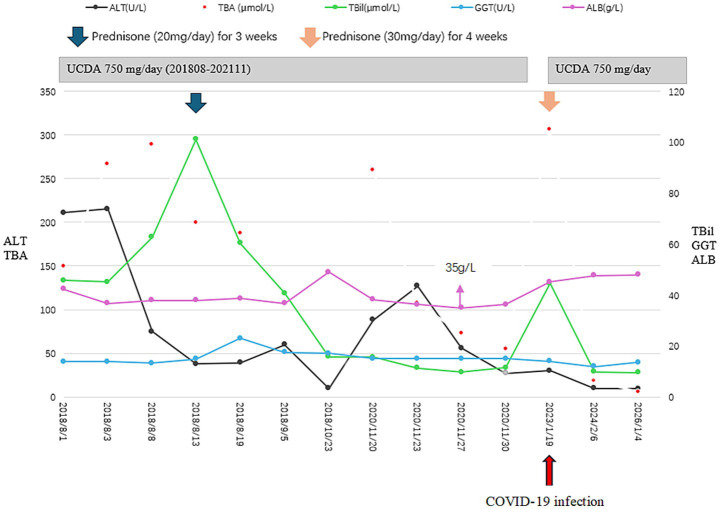
Clinical course of most significant laboratory tests in Case 2. GGT, Gamma-glutamyltranspeptidase; AST, Aspartate aminotransferase; ALT, Alanine aminotransferase. TBA, Total bile acid; TP, Total protein.

Following discharge, she continued regular administration of UCDA 250 mg three times daily. Two years later, she experienced another cholestasis attack and presented again to a local hospital. She was prescribed prednisone tablets 30 mg once daily as adjunctive therapy to UCDA, resulting in symptoms relief. After a year of continuous treatment, she discontinued medication due to pregnancy. Symptoms did not recur during her pregnancy. Four months postpartum, she developed the coronavirus disease 2019 (COVID-19) and subsequently experienced an attack, and was treated with UCDA 250 mg orally three times daily and prednisone tablets 30 mg orally once daily. Following symptoms resolution, prednisone was withdrawn, while UCDA was maintained. The condition has not recurred for nearly 3 years up to now. Figure 2 summarizes the most significant laboratory tests of her clinical course.

To further investigate the origin of ATP8B1 variants in two patients and to identify additional potential RIC1-affected individuals within the family, we performed ATP8B1 gene sequencing in all first-degree relatives across three generations and provided the pedigree of this family (Supplementary Figure 2). The results revealed that in two patients, the c.749 T > C variant was inherited from their mother, while the remaining six variants identified in Case 1 were entirely absent in the parents and were likely *de novo* events, and the c.3261 + 5G > A variant identified in Case 2 was paternally inherited. All seven relatives who underwent testing carried a heterozygous variant (c.3261 + 5G > A or c.749 T > C) of ATP8B1; however, none of them experienced similar cholestasis attacks observed in two patients.

## Discussion

RIC is a rare autosomal-recessive disorder characterized by recurrent attacks of icterus and itching with asymptomatic intervals. Its estimated incidence is approximately 1 in 50,000 to 100,000 people worldwide (7). Mutations in ATP8B1 and ABCB11 are responsible for RIC1 and RIC2 respectively, and are also involved in a PFIC type 1 and 2 characterized by a liver damage up to end-stage liver disease (11). To our best knowledge, this is the first case report presenting with novel ATP8B1 variants and genetic analysis of the whole family members within China, confirming the diagnosis criteria of RIC developed by Tygstrup (12).

Factors that precipitate attacks of RIC have been difficult to identify. In our report, Case 1 received antibiotic treatment prior to each of the three cholestasis attacks; however, the specific antibiotics administered and the clinical indications for their use remain undocumented. Cases of cholestasis attacks caused by antibiotic treatment have rarely been reported. To date, such an occurrence has only been observed in a South African adolescent with RIC (13). Although the precise mechanism underlying drug induced cholestasis has yet to be fully elucidated, accumulating evidence suggests involvement of hepatocellular transport dysfunction and immune-mediated injury (14, 15). These findings suggest that antibiotics should be used with extreme caution in RIC patients, particularly agents known to be closely associated with drug induced cholestatic liver injury such as amoxicillin/clavulanic acid (16).

A number of women have had cholestasis attacks during pregnancy or while taking oral contraceptives (17). The Case 2 did not experience an attack during any of her three pregnancies, most of attacks were secondary to exhaustion. However, an attack was following acute respiratory syndrome coronavirus-2 (SARS-CoV-2). It is assumed that cholestatic attacks generally begin after an upper respiratory tract infection (9). Post-mortem studies in patients with COVID-19 have shown bile duct proliferation and canalicular or ductular bile plugs in some patients, indicating the disease has a systemic course, including the liver (18, 19). Turan Çalhan et al. reported the only case of RIC attack triggered by COVID-19, hypothesized the underlying mechanisms and emphasized the significance of preventing COVID-19 infection in BRIC patients (20).

Histological findings of two patients revealed evidence of focal hepatocellular necrosis and hepatic fibrosis. Hepatocyte necrosis and inflammatory infiltration are among the less histological findings in RIC1 patients (21). Hepatic fibrosis is inherently a chronic and progressive pathological process. The Case 1 exhibited a refractory elevation in serum bilirubin levels, the GGT concentration was elevated to more than twice the upper limit of the reference interval (GGT levels in RIC patients are usually normal or only slightly elevated)during the sixth attack. A comprehensive evaluation was conducted to identify potential etiologies of hyperbilirubinemia and elevated GGT levels, including hemolytic disorders, viral hepatitis, autoimmune hepatitis, alcohol-associated liver injury and biliary tract disorders. However, no obvious causes were detected. Hypoalbuminemia was observed during the disease course of Case 2 (the lowest albumin concentration was 35 g/L). However, the albumin levels of RIC patients are usually elevated (22). Based on current clinical, biochemical and histological findings, the two patients’ clinical courses, differed from classical RIC1, emphasizing that recurrent cholestasis may not be entirely benign, which can be associated with mild histological abnormalities and clinical manifestations of varying severity relative to the prior attacks.

Useful lessons can be derived from the treatment outcome observed in Case 1. Given his concomitant hepatosplenomegaly and severe pruritus, the local hospital hypothesized an immune-mediated etiology and treated him with glucocorticoids and azathioprine. However, this regimen precipitated profound leukopenia, which subsequently culminated in fatal sepsis. No published literature has reported the use of immunosuppressants in the treatment of patients. Therefore, immunosuppressants should be avoided as much as possible in RIC patients, especially during their cholestatic attacks, which are recommended only when strict clinical indications are met. Further validation of the safety and efficacy of immunosuppressants is warranted through larger-scale, prospective studies. Recently, real-world studies have confirmed the efficacy of ileal bile acid transporter (IBAT) inhibition across different ATP8B1-related cholestatic phenotypes. Sprcifically, outside strict eligibility criteria, odevixibat is effective in reducing BA levels and improving pruritus (23).

ATP8B1 gene sequencing was performed on additional family members of the two patients to verify the familial inheritance pattern. Analysis revealed that apart from the two affected individuals, all other sequenced family members carried a heterozygous ATP8B1 variant and remained asymptomatic. Both two patients diagnosed with RIC1 were found to carry compound heterozygous variants of ATP8B1, consistent with the inheritance pattern of an autosomal recessive disorder. Indeed, a single heterozygous variant of ATP8B1 has been found in a single case of this disorder (1, 24–26), which has induced to hypothesize that a counter allele could be present but not detected, since common methods may not identify some variants (such as large deletions) and variants in untranslated regions or introns.

Among the seven variants of ATP8B1 identified in Case 1, the c.696 T > C, c.811A > C, and c.3454G > A have been previously reported in patients with familial intrahepatic cholestasis (13, 27, 28), whereas the remaining four variants and the c.3261 + 5G > A variant of ATP8B1 identified in Case 2 have not been described in the scientific literature and international databases. Pathological assessment reveals a marked reduction in ATP8B1 encoded protein expression in the liver tissue of Case 2. We believed that the two novel compound heterozygous variants of ATP8B1 in Case 2 are strongly associated with her clinical phenotypes. However, in Case 1, the available genetic data do not allow a definitive attribution of pathogenicity to specific variants, which illustrated the current limitations of genotype–phenotype correlations in ATP8B1-related disorders and aligned with the concept of considerable genetic and clinical heterogeneity highlighted in the recent literature (22).

To date, more than 554 variants are described in Clinvar database for ATP8B1 (ATP8B1[Gene]-ClinVar-NCBI. https://www. Ncbi. nlm. Nih. gov/clinvar/?term = ATP8B1% 5Bgene% 5D). The study of the genotype/phenotype relation is complicated by the presence of numerous variants. Additionally, hormonal factors linked to age, the presence of triggers, and ATP8B1 variants that produce a protein with reduced functionality make this investigation complex (22). A retrospective study showed a correlation between genotype severity and phenotype gravity in patients with ABCB4, ABCB11 and ATP8B1 variants. ATP8B1 variants were linked to chronic liver disease (75%); however, these variants, which had a low predicted pathogenicity, were common in patients with different underlying liver diseases (29). The two patients reported in this study, even within the same family, have different ages of onset and varying degrees of severity in their clinical manifestations. Moreover, given the absence of additional published cases with the same mutation sites as those of them, the genotype–phenotype correlations remain incompletely understood. Continued accumulation of well-characterized case reports and comprehensive analysis of the pathogenicity underlying ATP8B1 variants will be essential to clarify the clinical spectrum associated with ATP8B1 deficiency and to advance understanding of its underlying genotype–phenotype relationships.

In conclusion, we reported two rare siblings RIC1 cases with their atypical clinical courses and findings that different from classical RIC1 patients, providing valuable insights about factors that precipitate attacks of RIC and potential intermediate phenotypes between RIC1 and PFIC1. Notably, current evidence presented in this article suggests the potential pathogenicity of the two novel ATP8B1 variants (c.749 T > C and c.3261 + 5G > A), concurrently encouraging further investigations in the variability of ATP8B1 variants.

## Data Availability

The raw data supporting the conclusions of this article will be made available by the authors, without undue reservation.

## Supplementary material

SUPPLEMENTARY FIGURE 1 Variants of ATP8B1 identified in two patients through targeted next-generation sequencing. **(A)** A homozygous variant (c.696T > C, p.D232D) of ATP8B1 identified in the Case 1. **(B)** A heterozygous variant (c.+20C > T) of ATP8B1 identified in the Case 1. **(C)** A heterozygous variant (c.749T > C, p.L250P) of ATP8B1 identified in the Case 1. **(D)** A homozygous variant (c.811A > C, p.R271R) of ATP8B1 identified in the Case 1. **(E)** A heterozygous variant (c.2021T > C, p.M674T) of ATP8B1 identified in the Case 1. **(F)** A homozygous variant (c.3454G > A, p.A1152T) of ATP8B1 identified in the Case 1. **(G)** A heterozygous variant (c.3477C > T, p.P1159P) of ATP8B1 identified in the Case 1. **(H)** A heterozygous variant (c.749T > C, p.L250P) of ATP8B1 identified in the Case 2. **(I)** A heterozygous variant (c.3261 + 5G > A) of ATP8B1 identified in the Case 2.

SUPPLEMENTARY FIGURE 2 Pedigree and electropherograms of ATP8B1 variants identified in the family of the two BRIC patients through ATP8B1 gene sequencing. **(A)** A heterozygous variant (c.3261 + 5G > A) of ATP8B1 identified in the two patients' father. **(B)** A heterozygous variant (c.749T>C, p.L250P) of ATP8B1 identified in the two patients' mother. **(C)** A heterozygous variant (c.3261 + 5G > A) of ATP8B1 identified in the big sister of Case 2. **(D)** A heterozygous variant (c.3261+5G>A) of ATP8B1 identified in the second sister of Case 2. **(E)** A heterozygous variant (c.749T > C, p.L250P) of ATP8B1 identified in the son of Case 2. **(F)** A heterozygous variant (c.3261 + 5G>A) of ATP8B1 identified in the big daughter of Case 2. **(G)** A heterozygous variant (c.749T > C, p.L250P) of ATP8B1 identified in the second daughter of Case 2. **(H)** Pedigree of the family.
