# Supplementary figures and images for: Case Report: Recurrent intrahepatic cholestasis: two rare cases with their novel variants of ATB8B1 and atypical clinical findings
Source: Front Med (Lausanne). 2026 Jul 10;13:1886444. doi: 10.3389/fmed.2026.1886444 (PMC13395995; doi:10.3389/fmed.2026.1886444)

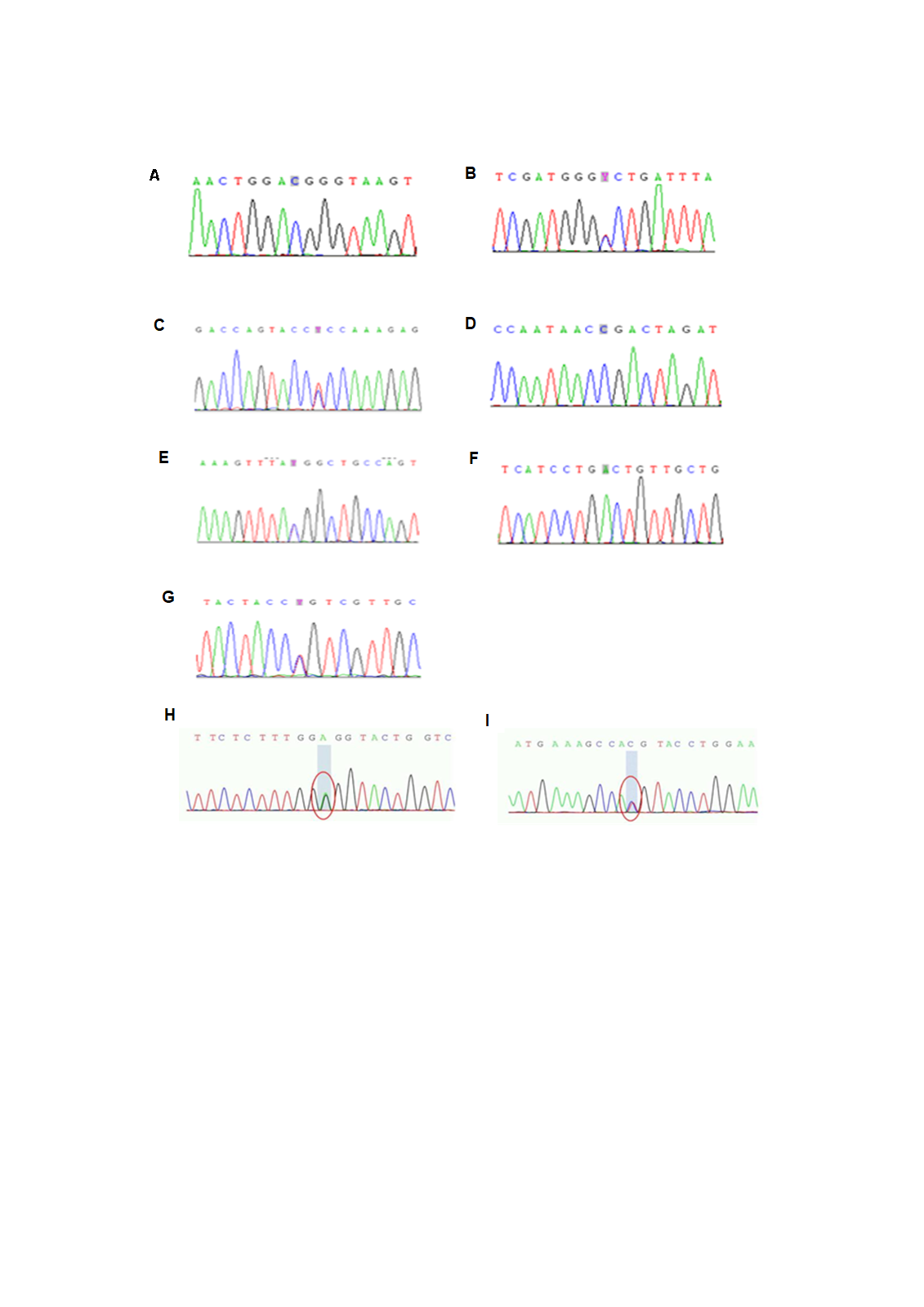

Supplement: SUPPLEMENTARY FIGURE 1 — Variants of ATP8B1 identified in two patients through targeted next-generation sequencing. (A) A homozygous variant (c.696T > C, p.D232D) of ATP8B1 identified in the Case 1. (B) A heterozygous variant (c.+20C > T) of ATP8B1 identified in the Case 1. (C) A heterozygous variant (c.749T > C, p.L250P) of ATP8B1 identified in the Case 1. (D) A homozygous variant (c.811A > C, p.R271R) of ATP8B1 identified in the Case 1. (E) A heterozygous variant (c.2021T > C, p.M674T) of ATP8B1 identified in the Case 1. (F) A homozygous variant (c.3454G > A, p.A1152T) of ATP8B1 identified in the Case 1. (G) A heterozygous variant (c.3477C > T, p.P1159P) of ATP8B1 identified in the Case 1. (H) A heterozygous variant (c.749T > C, p.L250P) of ATP8B1 identified in the Case 2. (I) A heterozygous variant (c.3261 + 5G > A) of ATP8B1 identified in the Case 2. [file Image_1.tif]

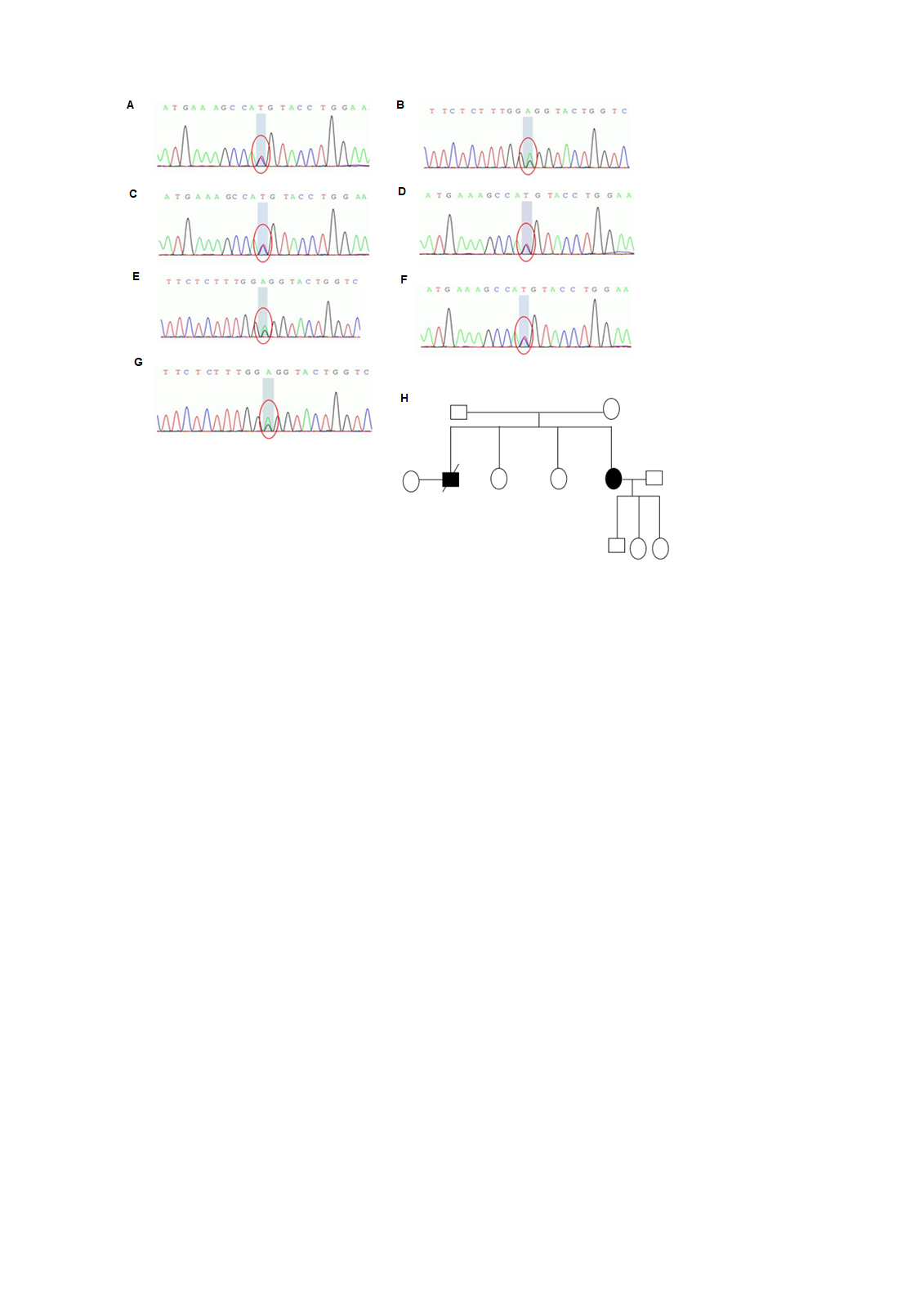

Supplement: SUPPLEMENTARY FIGURE 2 — Pedigree and electropherograms of ATP8B1 variants identified in the family of the two BRIC patients through ATP8B1 gene sequencing. (A) A heterozygous variant (c.3261 + 5G > A) of ATP8B1 identified in the two patients' father. (B) A heterozygous variant (c.749T>C, p.L250P) of ATP8B1 identified in the two patients' mother. (C) A heterozygous variant (c.3261 + 5G > A) of ATP8B1 identified in the big sister of Case 2. (D) A heterozygous variant (c.3261+5G>A) of ATP8B1 identified in the second sister of Case 2. (E) A heterozygous variant (c.749T > C, p.L250P) of ATP8B1 identified in the son of Case 2. (F) A heterozygous variant (c.3261 + 5G>A) of ATP8B1 identified in the big daughter of Case 2. (G) A heterozygous variant (c.749T > C, p.L250P) of ATP8B1 identified in the second daughter of Case 2. (H) Pedigree of the family. [file Image_2.tif]
