# Supplementary material for: Case Report: Recurrent intrahepatic cholestasis: two rare cases with their novel variants of ATB8B1 and atypical clinical findings
Source: Front Med (Lausanne). 2026 Jul 10;13:1886444. doi: 10.3389/fmed.2026.1886444 (PMC13395995; doi:10.3389/fmed.2026.1886444)
Supplement: Supplementary file 3 [file Data_sheet_1.pdf]

Supplementary table 1. Information of the ATP8B1 variants identified in two patients.

ATP8B1	Exon	DNA change	Protein	Zygosity	Reported/novel	Variant mutation	Source
Case 1	Ex8	c.696T>C	p.D232D	homozygous	Reported	Synonymous	De novo
		c.+20C>T	/	heterozygous	Novel	/	De novo
	Ex9	c.749T>C	p.L250P	heterozygous	Novel	Missense	maternal
	Ex10	c.811A>C	p.R271R	homozygous	Reported	Synonymous	De novo
	Ex18	c.2021T>C	p.M674T	heterozygous	Novel	Missense	De novo
	Ex27	c.3454G>A	p.A1152T	homozygous	Reported	Missense	De novo
		c.3477C>T	p.P1159P	heterozygous	Novel	Synonymous	De novo
Case 2	Exon	DNA change	Protein	Zygosity	Reported/novel	Variant mutation	Source
	Ex9	c.749T>C	p.L250P	heterozygous	Novel	Missense	maternal
	Ex24	c.3261+5G>A	/	heterozygous	Novel	Splicing	paternal
